# Post-Translational Modifications by Lipid Metabolites during the DNA Damage Response and Their Role in Cancer

**DOI:** 10.3390/biom12111655

**Published:** 2022-11-08

**Authors:** Guangrong Zhu, Xiangyang Zheng, Zhifeng Wang, Xingzhi Xu

**Affiliations:** 1 Guangdong Key Laboratory for Genome Stability and Disease Prevention, Carson International Cancer Center, Marshall Laboratory of Biomedical Engineering, Shenzhen University School of Medicine, Shenzhen 518060, China; 2 Guangdong Key Laboratory for Biomedical Measurements and Ultrasound Imaging, School of Biomedical Engineering, Shenzhen University School of Medicine, Shenzhen 518060, China; 3 Shenzhen University General Hospital-Dehua Hospital Joint Research Center on Precision Medicine, Dehua Hospital, Dehua, Quanzhou 362500, China

**Keywords:** DNA damage response, post-translational modification, lipid metabolites, acetylation, S-succinylation, palmitoylation, N-myristoylation, crotonylation, cancer

## Abstract

Genomic DNA damage occurs as an inevitable consequence of exposure to harmful exogenous and endogenous agents. Therefore, the effective sensing and repair of DNA damage are essential for maintaining genomic stability and cellular homeostasis. Inappropriate responses to DNA damage can lead to genomic instability and, ultimately, cancer. Protein post-translational modifications (PTMs) are a key regulator of the DNA damage response (DDR), and recent progress in mass spectrometry analysis methods has revealed that a wide range of metabolites can serve as donors for PTMs. In this review, we will summarize how the DDR is regulated by lipid metabolite-associated PTMs, including acetylation, S-succinylation, N-myristoylation, palmitoylation, and crotonylation, and the implications for tumorigenesis. We will also discuss potential novel targets for anti-cancer drug development.

## 1. Introduction

When genomic DNA is continuously exposed to harmful endogenous or exogenous assaults [1,2], DNA damage will invariably follow [3,4]. If this damage is not repaired properly, catastrophic adverse consequences can arise, limiting the adaptability and viability of cells and organisms [5,6]. Therefore, cells must act in a timely and effective manner to detect and repair any damaged DNA [7]. Cells have evolved a network of signaling pathways to deal with DNA damage, and these are collectively referred to as the DNA damage response (DDR). Different types of DNA damage require different repair mechanisms, such as mismatch repair (MMR) [8,9,10], base excision repair (BER) [11,12], nucleotide excision repair (NER) [13,14], and double-strand break (DSB) repair. The DDR typically follows these general principles: DNA damage leads to structural changes or the stagnation of DNA replication, which is then recognized by corresponding sensor proteins. These sensor proteins then recruit downstream effector proteins, which are responsible for initiating signal cascades and triggering cellular coping mechanisms, leading to the repair of damaged DNA. If the DNA damage is irreparable, the affected cells are likely to face the fate of being eliminated. However, if DNA damage is repaired improperly, the genome is left unstable, and somatic cells will become prone to various mutations. These include point mutations, insertions, deletions, and rearrangements, and may lead to unrestricted cell proliferation, malignant transformation and, ultimately, the occurrence and development of cancer. Therefore, an effective DDR is critical for the maintenance of genome stability and the prevention of cancer.

Protein post-translational modifications (PTMs) enrich the proteome through the covalent addition of functional groups to proteins, altering their function or localization [15]. There are >500 different types of PTM, including phosphorylation, methylation, acetylation, ubiquitination, ADP- ribosylation, and O-glycosylation, and these regulate both normal and pathological conditions in cells. Therefore, a comprehensive understanding of the range and effects of PTMs in cell biology, especially in terms of disease treatment and prevention, is of the utmost importance. In recent years, the in-depth study of protein functions and modifications through high-sensitivity mass spectrometry and related experimental techniques has led to the discovery of other intracellular metabolites, such as lactate, acetate, malonate, coenzyme-A, amino acids and lipids, which can also form covalent PTMs with proteins to regulate intracellular signal transduction. This review primarily focuses on covalent PTMs formed by lipid metabolites. The lipid metabolites described here can connect their hydrophobic groups to proteins through esters, thioesters, thioether bonds, and amides, completing the lipid modification of proteins. These modifications include acetylation, propionylation [16], malonylation [17], butyrylation [18], succinylation [19], palmitoylation [20], crotonylation [21], myristoylation [22], long-chain fatty acylation, acrylamidation and glycosylphosphatidylinositol anchoring [23], 2-hydroxyisobutyrylation [24] and β-hydroxybutyrylation [25]. A majority of acyl-CoA species for PTMs are associated with metabolic processes in mitochondria directly or indirectly involved with the β-oxidation of fatty acids [26,27]. However, it is well established that acyl-CoAs are not directly exported across mitochondrial membranes. They must be synthesized outside of mitochondria [27]. On the other hand, short-chain fatty acids (SCFA), such as acetate, crotonate, butyrate, and propionate, are converted to acyl-CoA by the acyl-CoA synthetase short-chain family member 2 (ACSS2) [28]. These protein-lipid modifications promote the stable binding of proteins and membranes through the covalent binding of small, hydrophobic molecules and thus regulate protein transport and other protein-protein interactions. Notably, certain lipid modifications are thought to be involved in the DDR process, which is critical to maintaining the stability and integrity of the genome. In this review, we will summarize the spatiotemporal regulation of the DDR by these PTMs and discuss the implications in terms of tumorigenesis and anti-cancer drug targets.

## 2. Acetylation

### 2.1. Acetylation of Histones and Non-Histone Proteins

In eukaryotic cells, chromatin refers to a linear, complex structure composed of histone, non-histone, DNA, and a small amount of RNA and is located in the nucleus. Histones are alkaline proteins that bind to DNA in eukaryotic cells. They are composed of four core histone families (H2A, H2B, H3, H4) and a linker family (H1). Core histones are mainly responsible for binding to DNA to form nucleosomes, which represent the structural units of chromatin, while linker histones are responsible for binding with nucleosomes to form the next chromatin unit. Thus, histones play an important role in maintaining chromatin structure. Non-histone chromatin refers to other proteins that can bind to chromatin, including chromatin structural proteins, enzymes, and a small number of regulatory proteins. Although non-histones are less common in chromatin than histones, there a wide range of types, and they serve complex functions, such as regulating gene expression and maintaining chromatin structure. There are more basic amino acids in histones than acidic amino acids, but the ratio of basic to acidic amino acids in non-histones varies across situations and contexts. 

**Figure 1:**
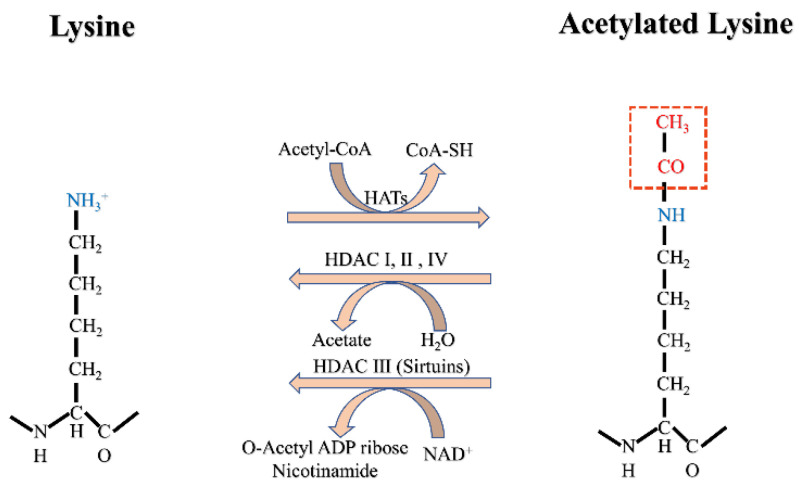
Acetylation modification mechanism.

Histone acetylation is a dynamic and reversible protein PTM and may be the most well-understood. Histone acetyltransferases (HATs, also referred to as lysine acetyltransferases) and histone deacetylases (HDACs, also known as lysine deacetylases) catalyze the addition or removal of acetyl groups, respectively, to lysine residues on both histone and non-histone proteins [29]. Acetylation occurs on amino-terminal proteins and on the O-junctions of serine and threonine, but for the purposes of this review, acetylation refers only to N ε-lysine acetylation (Kac) (defined as the deposition of an acetyl group onto the epsilon amino group of lysine; Figure 1) [30]. It was found that, by stable isotope tracking and acetylation proteomic analysis, more than 90% of acetylation modification on histone lysine is derived from the carbon of fatty acids [26]. Acetyl-CoA in mitochondria from fatty acid β-oxidation is converted to citrate, an intermediate of the tricarboxylic acid cycle. Citrate is exported out of mitochondria by citate transporter and subsequently cleaved by ATP-citrate lyase (ACLY) into acetyl-CoA [31]. 

Acetylation of histones by HATs relaxes the structure of chromatin and increases DNA accessibility, which results in increased gene transcription. The effect of HDACs is the opposite, with deacetylation making the structure of chromatin more compact and inhibiting transcription. The histone code hypothesis suggests that histone modification can be explained by chromatin-binding proteins called “readers,” which distinguish between modified and unmodified nucleosomes and then decide on DNA transcription or other related downstream events [32]. At present, known domains with the ability to recognize or read acetylated lysine residues include YAF9, ENL, AF9, Taf14, and Sas5 YEATS domains, and tandem homologous domains (PHDs, also known as double PHD finger (DPF) domains) in plants [33]. In addition to histones, mass spectrometry has also revealed that acetylation occurs on non-histone proteins, some of which participate in protein folding, protein degradation, and chromatin structure adjustment [34].

The exact number of HATs in the human proteome remains unknown. However, the histone HATs that have been identified thus far include the P160, P300/CBP, TAFII230, MYST, GNAT, and PCAF families, with the current classification system primarily being based on homology with the original enzyme sequence found in yeast. In addition, a total of 13 non-histone HATs have also been identified, and these are roughly divided into three families: GCN5, P300, and MYST. These include histone acetyltransferase 1 (HAT1, also known as KAT1), α-tubulin N-acetyltransferase 1 (TAT1, also known as ATAT1) [35], and establishment of cohesion 1 homology 1 (ESCO1) and ESCO2, which all serve different functions. At present, non-histone HAT specificity is thought to be determined by the accessibility of lysine in their substrate proteins, specific subcellular localization, and interacting proteins. For example, with the exception of TAT1, HATs are primarily located in the cell nuclei. Most HAT substrates do not overlap, but some functionally similar HATs can acetylate the same sites: for example, CREB-binding proteins (CBP, also known as KAT3A) and p300 (also known as KAT3B) both acetylate histone H3Lys18 (H3K18) and H3K27.

HDACs, meanwhile, are divided into four categories based on their sequence similarity and degree of phylogenetic conservation. The class I HDACs include HDAC1, 2, 3, and 8; the class II HDACs include HDAC4, 5, 6, 7, 9, and 10 (being further subdivided into class IIa and IIb); the class III HDACs are known as the sirtuins and include SIRT1-7; while the fourth category currently includes HDAC11 only [36]. With the exception of class III HDACS, which are NAD^+^-dependent, the other HDAC classes are all zinc-dependent. Zinc-dependent HDACs contains a deacetylase domain which is highly conserved and are often referred to as classical HDACs. While class I and IV HDACs are localized in the nucleus, class IIb HDACs are distributed in the cytoplasm. Class IIa HDACs are mainly distributed in the nucleus but are exported to the cytoplasm when activated. The sirtuins are distributed in different locations in the cell as follows: cytoplasm (SIRT2), mitochondria (SIRT3, SIRT4, and SIRT5), nucleus (SIRT1 and SIRT6), and nucleolus (SIRT7).

It is worth noting that most non-histone deacetylases have limited or no deacetylase activity or primarily perform other types of acylation. For example, SIRT4 [37] removes the acyl group from hydroxymethyl glutaryl lysine, SIRT5 [38] acts as a demalonylase and a deglutarylase, and SIRT6 acts as a long-chain fatty acid deacylase. Class IIa HDACs lack obvious catalytic activity, primarily due to the alterations in the conserved amino acids found in their catalytic pocket. Recent studies have found that lymphoid augmenter factor 1 (LEF1) [39] and T cell-specific transcription factor 1 (TCF1, also known as Tcf7) [40] also exert HDAC activity and that these two transcription factors are involved in the regulation of the WNT signaling pathway [41]. The overall sequences of LEF1 and TCF1 are very different from HDAC8, but their functions are similar. 

Recent studies have shown that acetylation can also occur through non-enzymatic mechanisms [42]. For example, lysine can be acylated by acyl-CoAs formed by the breakdown of fatty acids, and this mostly occurs through non-enzymatic mechanisms. Interestingly, some acyl-CoAs, such as glutaryl coenzyme A and succinyl coenzyme A, are derived from the carboxy cycle process but are more active than acetyl-CoA. The primary mechanism underpinning these modifications is acyl-CoA carboxyl group-induced intramolecular nucleophilic attack on CoA thioester bonds, which results in the formation of cyclic anhydride. This is more active than acyl-CoA and can produce non-enzymatic modifications more efficiently. Non-enzymatic acylation is thought to be affected by the cellular concentration of acyl-CoAs, the reactivity of acyl-CoA, local pH levels, and the number of lysine residues in proteins. These factors may differ between cell and tissue types. However, the specific regulatory mechanisms of this non-enzymatic modification remain unclear and require further investigation. 

### 2.2. Acetylation in the DDR

#### 2.2.1. Histone Acetylation in the DDR

Histone acetylation is well known to lead to changes in the structure of chromatin. Mechanistically, this occurs in two ways: on the one hand, the positive charge of lysine residues can be neutralized after acetylation, resulting in the weakening of the interaction between histone and DNA skeleton and the promotion of chromatin decompaction. This exposes more sites and enhances the accessibility of nucleosomal DNA. On the other hand, related chromatin remodeling complexes, such as SWI/SNF complexes, can also be recruited to the chromatin region to regulate chromatin structure. It was found that, in response to ionizing radiation (IR), nuclear ACLY is phosphorylated at S455 by ataxia telangiectasia mutated (ATM) and facilitates histone acetylation at DSBs, promoting HR-mediated repair by enabling BRCA1 recruitment while impairing 53BP1 recruitment [43]. This direct evidence demonstrates the link between histone acetylation and DDR. Other studies have shown that the acetylation modification of histone H1K85 can mediate chromatin changes under the dynamic regulation of acetylase PCAF and deacetylase HDAC1 in response to DNA damage, thus ensuring genome stability [44]. However, compared with H1 histone, H2 histone acetylation has been studied more extensively in terms of DDR. When cells are stimulated by ionizing radiation (IR), H2AX lysine 36 (H2AX K36ac) can be acetylated by acetyltransferase CBP/P300 to recruit corresponding DNA repair proteins to DNA damage sites [45]. Furthermore, under ionizing radiation-induced stimulation, the DNA PKCs BRD domain can specifically recognize H2AX acetylated lysine 5 (K5ac), which can lead to the determination of cell fate via γH2AX [46]. Moreover, under IR stimulation, knockdown of the tumor suppressor gene ZNF668, known to be involved in breast cancer, will weaken the interaction between Tip60 and H2AX. This leads to reduced hyperacetylation of histone H2AX and the prevention of chromatin relaxation, resulting in a reduced recruitment of repair proteins to DNA damage sites, defective homologous recombination (HR) repair, and reduced cell survival rates. These examples fully illustrate the importance of H2 histone acetylation to the DDR and, thus, to cancer [47].

Histone H3 acetylation also serves an important regulatory role in the DDR. At the location of DNA damage, the histone deacetylases HDAC1 and HDAC2 maintain low H3K56 acetylation levels to ensure that the damaged DNA is repaired. After the repair is completed, the proteasome will degrade the acetylated core histones, and the newly formed core histones will be assembled into nucleosomes. This represents the completion of the process of coping with DNA damage and repair [48]. PhD bromo tandem domain containing trimer motif 66 (TRIM66) has also been found to recognize unmodified H3R2, H3K4, and acetylated H3K56, while TRIM66 can recruit SIRT6 to deacetylate H3K56, thereby initiating the DDR and maintaining genome stability [49]. In addition, after 24 h of stimulation of HaCaT cells with sodium arsenite (NaAsO_2_), arsenic has been reported to reduce histone H3K18 acetylation levels, affect the expression of xeroderma pigmentosa-related proteins (XPA, XPD, and XPF nucleotide excision repair (NER)-related genes), and further aggravate DNA damage. However, the use of histone deacetylase inhibitor trichostatin A (TSA) can inhibit the deacetylation of H3K18 in the promoter region of XPA, XPD, and XPF, increase the acetylation of H3K18, and promote the transcriptional expression of NER-related genes. To a certain extent, it can inhibit arsenic-induced DNA damage [50].

In addition to H1, H2, and H3 acetylation, some studies also have been conducted on histone H4 acetylation. After doxorubicin treatment, cells in G0/G1 phase experience DNA damage but it can also promote the activation of chromatin kinase VRK1. VRK1 directly interacts with Tip60 and phosphorylates it, resulting in increased histone H4K16 acetylation, a marker of local chromatin relaxation. Inhibition of Tip60 expression by siRNA or its kinase inhibitor MG149 inhibited H4K16 acetylation, indicating VRK1-mediated phosphorylation of Tip60 increases its enzymatic activity. This work suggests that the dynamic remodeling of chromatin is closely related to the epigenetic modification of histone [51]. In addition, males absent of the first (MOF) proteins can regulate the level of H4K16 acetylation. MOF is responsible for maintaining sufficient levels of H4K16 acetylation in cells, which facilitates the generation of chromatin structures conducive to DNA repair. When MOF is absent in cells, the acetylation level of H4K16 is reduced and ultimately, the recruitment and cancellation of the corresponding signal proteins at the DNA damage site is impaired [52]. Furthermore, after DNA double-strand breaks induced by the HO endonuclease system, if the acetylation sites of newly synthesized histone H4 are mutated, the reassembly of chromatin structure is inhibited. Interestingly, the newly synthesized histone H4 acetylation mutation site changes, resulting in phosphorylated H2A (γ-H2AX) levels significantly decreasing around DSBs, indicating the critical role of chromatin assembly in DNA damage signaling [53].

In summary, Kac modification of histone tails can lead to the relaxation of chromatin structure, which is more conducive to the completion of DNA repair after DNA damage. Moreover, after DNA repair is completed, histones undergo deacetylation, and the chromatin structure becomes compact. Thus, this dynamic regulation by histone acetylation is critical to the maintenance of genome stability.

#### 2.2.2. Non-Histone Acetylation in the DDR

Non-histone acetylation has also been found to participate in the DDR process. When inducing DNA damage in human cells, the acetylation of lysine 382 and phosphorylation of serine 392 in p53, a key DDR factor, can significantly enhance the interaction between p53 and MDC1 and promote the recruitment of these two proteins to DNA damage sites [54]. In response to DNA damage, N-acetyltransferase 10, NAT10 (also known as HALP), a member of the GNAT family, translocates to the nucleoplasm, promoting p53 acetylation at K120 with its acetyltransferase activity and proteosome-mediated degradation of MDM2 with its intrinsic E3 ligase activity, ultimately resulting in stabilizing p53 and p53-mediated cell cycle arrest and apoptosis [55]. Tip60-mediated acetylation, the DDR core kinase ATM also activates its kinase activity and subsequent checkpoint signaling upon DNA damage, while Tip60 inactivation sensitizes cells to ionizing radiation [56].

Werner syndrome is a rare autosomal recessive disease caused by mutations of the *WRN* gene. When the lysines K1127 and K1117 of WRN are mutated to arginine, cells may become sensitive to DNA-damaging agents such as mitomycin C and etoposide, indicating defective DNA repair. In fact, these two sites are critical for the recruitment of WRN to DNA damage sites [57].

Furthermore, the acetylation of proteins has also been found to be involved in the regulation of base excision repair (BER). For example, acetylation of depuridine/depyrimidine endonuclease 1 (APE1; also known as APEX1), an important regulator of BER, inhibits its interaction with XRCC1 when DNA damage occurs; resulting in decreased APE1 activity. However, SIRT1 can deacetylate APE1, recovering its function [58]. Additional acetylated non-histone proteins involved in the DDR are summarized in Table 1.

#### 2.2.3. Roles of HATs and HDACs/SIRTs in DDR

Many HATs-mediated acetylations of histone and non-histone proteins directly or indirectly modulate DDR. KAT8 (hMOF), as a member of the histone acetyltransferase MYST family, can respond to DNA damage by catalyzing the acetylation of H4 at K16 (H4K16) and p53 at K120 [59,60]. KAT5 (TIP60) is also a member of the histone acetyltransferase MYST family [61], and its regulatory role in DNA damage signaling [62] has been reported. It was found that TIP60 can regulate the acetylation of a variety of histones (H2, H3, and H4) and non-histones (p53 [63] and ATM [64]). For example, TIP60 can acetylate the K15 position of H2A in response to DNA damage [65,66]. It has also been shown that Tip60 regulates the acetylation of H4 by forming a complex with transformation/transfer domain-associated protein (Trrap) and then promotes DNA damage repair by HR [67]. In addition, histone acetyltransferases KAT3A (CBP) and KAT3B (p300) are structurally similar and participate in the regulation of many functions in cells. In vitro enzyme activity test showed that CBP/p300 could acetylate all acetylation residue sites of histone H2A and H2B, among which K14, K18, and K56 of H3 and K5 and K8 of H4 were preferentially oxidized [68]. Moreover, CBP can also regulate the acetylation of non-histones in cancer. In human colon cancer cells, CBP can mediate the acetylation of K358 of DOT1L, which is positively correlated with the staging of colon cancer [69]. It was found that in vitro, acetyltransferase GCN5 can form a complex with its chaperone PCAF to acetylate multiple lysine residue sites on histone H3, such as H3K9, H3K14, H3K18, and H3K23 [70,71,72,73]. In addition, PCAF can acetylate H1K85 in response to DNA damage [74].

On the other hand, HDACs/SIRTs-mediated protein deacetylation also plays an important role in DDR. In mammals, SIRT1-7 has different subcellular localization, functions, and substrates and was initially identified as histone and non-histone protein deacetylases [75,76]. It was found that sirtuins can mediate the specific deacetylation of histone lysine residues to facilitate DNA repair. In mammals, H3K56 will undergo hyperacetylation upon inhibition of SIRT1 expression, leading to the instability of the S phase genome [77]. It has also been reported thatSIRT3, which is mainly located in mitochondria, will be transported to the nucleus to regulate the deacetylation of H4K16ac in response to DNA damage [78,79,80]. SIRT6 is an intranuclear deacetylase which plays an important role in regulating DDR signal and genome stability. Its histone substrates include H3K9, H3K18, and H3K56. H3K9 deacetylation mediated by SIRT6 can protect telomeres in mammalian cells. On the contrary, the lack of SIRT6 will lead to chromosome fusion due to telomere dysfunction [81]. Similarly, in the nucleus, SIRT7 can catalyze the deacetylation of H3K18ac at the late response to IR [82]. In addition, HDAC1/2-mediated deacetylation of H3K56 and H4K16 also plays an important role in chromatin regulation [83,84]. It was found that histone deacetylase was rapidly recruited to the DNA damage site, leading to histone deacetylation, thereby promoting non-homologous end joining (NHEJ) repair [83]. Several studies have found that the levels of H3K56ac, H4K16ac, and H4K91ac will increase after HDAC1 expression is inhibited, which is related to the decreased cell survival rate after treatment with DNA damaging reagents [83,84,85,86].

In conclusion, the dynamic regulation of both histone and non-histone acetylation and deacetylation serves an important function in the repair of DNA damage.

**Table 1:** Lipid metabolite associated PTMs in the DDR.

Modification	Writers	Erasers	Readers	Substrates in the DDR	References
Acetylation (histone)	P160, P300/CBP	HDAC1-11,	YAF9, ENL, AF9, Taf14,	H1K85	
	TAFII230, MYST,	SIRT1-7	Sas5(Yeats),	H2AX K5, K36	[44,45,46,49,50,51]
	GNAT, PCAF		PHDs	H3K4, K18, K56 H4K16	
Acetylation (non-histone)	GCN5, P300, MYST19,	HDAC1-11		Tip60, APE1,	
	KAT1, TAT1, ESCO1-2	SIRT1-7, LEF1, TCF1	NA	PFKFB3 K472, OGG1, Cdc25A, P53K382/K120, WRN K1117/K1127, MLH1, RRM2 PARP1 K949,	[34,87,88,89,90,91]
Succinylation	NA	KDAC: SIRT5, SIRT7	NA	H3K12, P53K120, H4K77,	
				ACOX1(acyl-CoA oxidase 1), FEN1 K200, NPM1	[19,92,93,94,95,96]
Palmitoylation	DHHC1-23	APT1-2	NA	Rap1-interacting factor 1(Rif1) C466/C473	[97]
N-myristoylation	NMT1-2	SIRT1-3, SIRT6	NA	Finkel-Biskis-Reilly (FBR) v-fos	[98]
Crotonylation	P300/CBP, MOF	HDAC1-3, SIRT1-3	YEATS, PHD	RPA1	[21]

Abbreviations: NA, not available; ESCO1, the establishment of cohesion 1 homolog 1; PFKFB3, phosphofructokinase-2/fructose-2,6-bisphosphatase 3; WRN, Werner syndrome protein; MLH1, MutL Homolog 1; RRM2, Ribonucleoside-Diphosphate Reductase Subunit M2; FEN-1, flap endo/exonuclease; RPA1, Replication factor A protein 1; KAT, lysine (K) acetyltransferase family; DHHC, aspartate–histidine–histidine–cysteine family; NMT, N-terminal myristoyltransferase family.

### 2.3. Acetylation in Cancer

#### 2.3.1. HATs and Cancer

HATs are well known to be involved in tumorigenesis and cancer progression, with HAT activity being altered either by gene mutations or viral oncogenes in both blood and solid cancers. For example, the interaction between adenovirus SV40T antigen protein E1A and the co-activators p300 and CBP play a key role in cell transformation [99]. These HATs are then redistributed to the promoter regions of certain genes to promote cell growth, differentiation, and the transcriptional activation of specific genes [100]. Ambiguous p300 mutations can be found in solid stomach, rectum, breast, and prostate tumors [101,102,103].

Tip60 is another HAT that is closely associated with tumorigenesis [104,105,106] and may be involved in the regulation of DNA repair and the transcriptional activation of p53 and Myc [62,107]. Decreased expression of Tip60 results in low p53 acetylation levels and incomplete apoptosis signaling, which indicates transformation to a malignant tumor [108]. As a tumor suppressor protein, single allele deletion of human Tip60 is often found in head and neck tumors, breast cancer, and lymphoma [109]. In addition, Tip60 has also been found to inhibit Myc-mediated lymphoma formation in B-cell lymphoma [110].

#### 2.3.2. HDACs and Cancer

HDACs function in the opposite manner to HATs, regulating transcription by removing acetyl groups from lysine residues of histone tails and other non-histone substrates. Thus, it stands to reason that they would also be involved in cancer. Functional experiments have indicated that type I HDACs are mainly responsible for regulating cell proliferation and apoptosis, while type II HDACs regulate cell metastasis and angiogenesis. For example, inhibiting HDAC1 and HDAC2 in vitro can inhibit colon cancer cell proliferation [111,112]. However, the inhibition of HDAC3 suppresses the proliferation of colon cancer cells more substantially [113]. In addition, inhibition of HDAC2 and HDAC3 is known to induce DDR and apoptosis after DNA damage.

Type II and IV HDACs are primarily localized in the cytoplasm and are mainly responsible for the deacetylation of non-histone proteins. Previous studies have shown that inhibiting HDAC4 reduces colon cancer cell proliferation and induces apoptosis [114,115,116]. Furthermore, while the inhibition of HDAC7 in endothelial cells does not affect cell growth and survival, it does inhibit cell metastasis and the formation of capillary-like structures in cancer [117,118]. Type II HDACs are mainly responsible for regulating angiogenesis, and inhibition of HDAC6 and HDAC10 results in decreased VEGFR1 and two expressions [119].

Type III HDACs, also known as the sirtuins, share no sequence homology with other deacetylases. However, they may also be involved in regulating the occurrence and development of tumors. Sirtuins induce the deacetylation of a range of protein substrates, including histones, but also mediate ADP ribosylation. Furthermore, overexpression of SIRT1, 2, 3, and 7 have been identified in many types of cancer [120,121,122]. For example, overexpression of SIRT1 is known to prevent apoptosis by regulating histone deacetylation, promoter methylation, and histone methylation and inhibiting the transcription of tumor suppressor genes. This ultimately promotes cancer cell growth by preventing cell senescence and differentiation, as well as the formation of tumor blood vessels by promoting the growth of endothelial cells and preventing their aging [123]. Interestingly, the expression of SIRT2 is absent in human glioma cells, and re-expression of SIRT2 can reduce the ability of colony-stimulating factor formation of cells [124]. This suggests that, in some cases, Sirtuins also serve as tumor suppressors. At present, whether Sirtuins function as oncogenes or as tumor suppressors remains controversial. However, it is clear that altered HDAC function plays a corresponding role to that of HATs in the process of tumorigenesis and development.

#### 2.3.3. Summary

The extensive research conducted thus far on protein acetylation and the fact that abnormal acetylation is closely associated with cancer has laid the foundation for the discovery of many novel epigenetic drug targets. Certain HDAC inhibitors have been approved for cancer treatment, including romidepsin, panobinostat, and belinostat, while more are being tested in clinical trials. However, most research exploring the potential for HAT inhibitors to treat cancer is yet to enter clinical trials, leaving this treatment avenue in its primary stages. However, the identification of new HAT subtypes and improved characterization of their roles and functions have provided more potential treatment strategies. For example, curcumin, as a natural KATi, can inhibit the activity of p300/CBP and so suppress the proliferation of a variety of cancer cells, thereby achieving anti-inflammatory and anti-tumor effects [125]. Moreover, as a small molecule derived from anacardic acid, MG153 also acts as a potential p300/PCAF inhibitor and can suppress the proliferation of BCR-ABL-expressing cells, induce apoptosis, and resist DNA damage [126]. In addition, L002, a small molecule inhibitor of p300, has been shown to inhibit p300, PCAF, and GCN5 activity in leukemia, lymphoma, and breast cancer cell lines [127]. In conclusion, further research on both HDAC and HAT inhibitors will likely prove very fruitful when developing novel treatments for cancer [128].

## 3. Succinylation

### 3.1. Succinylation

**Figure 2:**
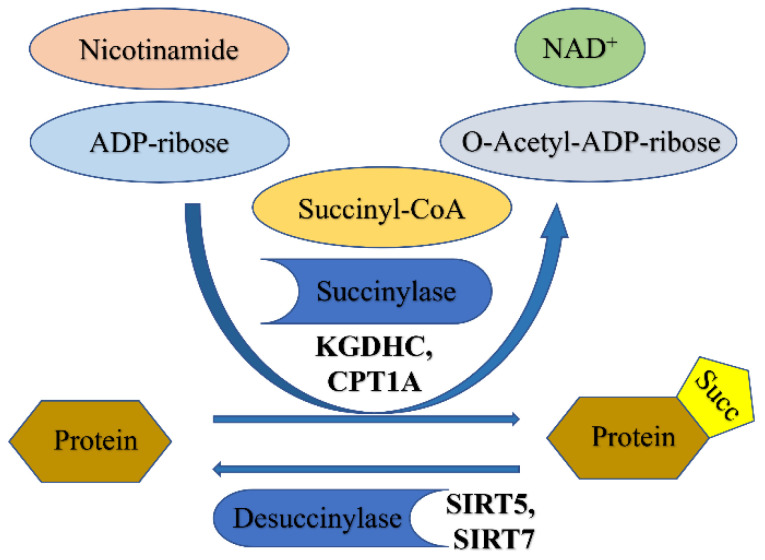
Succinylation modification mechanism.

Lysine succinylation (KSucc) is a newly discovered PTM, and relatively few studies have been published thus far [129,130,131]. Both succinate and α-ketoglutarate, two intermediates of the TCA cycle, are transported out of mitochondria with the help of transporters and converted into succinyl-CoA by α-ketoglutarate dehydrogenase complex and an unknown mechanism, respectively [27]. Succinyl-CoA serves as a donor for succinylation. The underlying mechanism of this modification is as follows: lysine side chains have a positive charge and so participate in non-covalent interactions with negatively charged residues, such as hydrogen bonds and electrostatic interactions. KSucc refers to the combination of lysine side chain residues with succinate group (-CO-CH_2_CH_2_CO_2_H), which lends the side chain a negative charge and significantly impacts protein function. Recent developments in high-throughput proteomic techniques, combined with PTM analysis, have revealed that lysine succinylation modifications frequently occur in the cytoplasm, mitochondria, and nucleus and are responsible for regulating a range of different physiological functions in cells. These functions include fatty acid synthesis, fatty acid oxidation, and mitochondrial respiration [132]. The succinylation process is illustrated in Figure 2.

#### 3.1.1. Enzymatic Regulation of Succinylation

Lysine succinyltransferases (KSTases) are responsible for catalyzing lysine succinylation. For example, lysine acetyltransferase 2A (KAT2A) is known to succinylate H3K79 and to promote tumor cell proliferation [133]. Succinyl CoA is also known to be produced when tyrosine 645 (Y645) in KAT2A interacts with α-ketoglutarate dehydrogenase complex (a-KGDH) in the nucleus [134]. Failure of aKGDH to enter the nucleus or mutations in Y645 in KAT2A to nonfunctional alanine prevents the succinylation of H3K79 and inhibits tumor cell proliferation [134].

Another previous study has also demonstrated that carnitine palmitoyltransferase 1A (CPT1A), a mitochondrial outer membrane protein oxidized by fatty acids, has significant KSTase activity [135,136,137]. In fact, a total of 171 lysine sites on 101 proteins have been found to be succinylated in a CPT1A-dependent manner [136]. Notably, the classical CPTase activity of CPT1A can function in tandem with the newly discovered KSTase activity. However, when the G710E mutation occurs in CPT1A, these two activities become discrete. This leads to the promotion of cell proliferation under metabolic stress but does not affect intracellular succinyl CoA levels. This suggests that the KSTase activity of CPT1A promotes cell proliferation through the succinylation of downstream substrate proteins [16].

#### 3.1.2. Non-Enzymatic Succinylation

KSucc has also been found to occur through non-enzymatic chemical reactions, such as those mediated by succinyl CoA. Previous studies have shown that succinyl coenzyme A is particularly rich in the mitochondria, suggesting that the succinylation of proteins in the mitochondria occurs via non-enzymatic mechanisms [138]. A study aiming to confirm this found that the loss of succinate dehydrogenase (SDH) and the continuous accumulation of succinate CoA resulted in excessive lysine succinylation accumulating in multiple cell septa, causing TCA circulation dysfunction [139]. This demonstrated that both cytoplasmic and nuclear proteins could produce succinyl CoA. Another study found that R132H mutations in nicotinamide adenine dinucleotide phosphate isocitrate dehydrogenase (NADP-IDH) were associated with a significant increase in cellular succinyl-CoA levels and hyper-succinylation in the mitochondria. This ultimately led to respiratory disorders in the mitochondria [140]. In summary, succinyl coenzyme A promotes succinylation by non-enzymatic mechanisms, providing a broader framework for the study of succinylation.

#### 3.1.3. De-Succinylation

Sirtuin5 (SIRT5) is an HDAC and is primarily known for its ability to deacetylate lysine. However, in recent years, SIRT5 has also been found to exhibit de-succinylation activity [141,142]. SIRT5 was initially shown to deacetylate and upregulate the activity of carbamyl phosphate synthase 1 (CPS1) during the regulation of the urea cycle [143]. CPS1 K1291 succinylation levels were also found to increase significantly in SIRT5-knockout mice, which confirmed that SIRT5 could regulate KSucc levels in vivo [144].

SIRT5 was originally thought to be located exclusively in the mitochondria. However, more recent data have demonstrated that SIRT5 also serves a regulatory role in the cytoplasm. Deletion of SIRT5 in the mitochondria leads to the hypersuccinylation of various proteins, including CPS1 and other enzymes associated with fatty acid metabolism. Furthermore, SIRT5 knockout mice exhibit high succinylation of numerous proteins involved in the main metabolic pathways, such as ATP synthesis, TCA cycle, and fatty acid β- oxidation, compared with WT mice. Taken together, these results indicate that SIRT5 plays an important role in the regulation of KSucc in addition to its deacetylase function.

#### 3.1.4. Lysine-Succinyl Readers

At present, the research on lysine succinyl readers is very limited. The Yeats domain is only found in glioma-amplifiedsequence-41 (GAS41) [145] and binds to succinylated histones in a pH-dependent manner. However, at present, no brominated domain with an affinity for succinyl lysine has been found. Subsequent studies need to be performed to identify more readers of lysine succinylation [27].

### 3.2. Succinylation in the DDR

SIRT7, as a deacetylase, promotes the inactivation of ataxia-telangiectasia mutated (ATM) via its catalytic deacetylation modification function [146]. However, during DNA damage, SIRT7 is also recruited to DNA damage sites in a poly (ADP) ribose polymerase 1 (PARP1)-dependent manner to catalyze H3K122 desuccinylation. This increases the binding of histone 3 to DNA, which aids chromatin condensation and repair after DNA damage. However, when there is a lack of SIRT7 in the cells, the desuccinylation of H3K122 is reduced, and thus, the DDR is inhibited [147]. This prompted an idea for a novel follow-up study on the role of histone desuccinylation in the regulation of gene expression [93]. In a similar vein, a recent study has shown that lysine-glutamate mutations at the H4K77 site, mimic de-succinylation, reduce nucleosome stability, and prevent the repair of damaged DNA [19]. Furthermore, p53 serves as a common tumor suppressor in cells and serves an important role in the maintenance of genomic stability. It can be succinylated at lysine 120 (K120) and is desuccinylated by SIRT5, which results in the inhibition of p53 activity and attenuates its maintenance of genomic stability [92].

In mammals, acyl CoA oxidase 1 (ACOX1) is a rate-limiting enzyme in the peroxisomal pathway, which catalyzes the oxidation of very-long-chain fatty acids in the peroxisome to produce a large amount of hydrogen peroxide. It has been reported that ACOX1 dysfunction is often closely related to peroxisome diseases and liver cancer. It was found that peroxisomes contain deacetylase sirtuin 5 (SIRT5) [94]. Under physiological conditions, SIRT5-mediated de-succinylation of ACOX1 inhibits its oxidase activity, thereby reducing the production of hydrogen peroxide, weakening DNA oxidative damage, and ultimately ensuring genome integrity.

While post-translational histone modifications are known to be involved in the regulation of nucleosome and chromatin kinetics, the newly discovered role of Ksucc in these processes remains poorly defined. The H4K77 succinylation site is highly conserved at the DNA-histone interface of the nucleosome, and the succinylation of H4K77 results in DNA relaxation. In budding yeast, mutations that substitute lysine with glutamic acid in the H4K77 succinylation site have been found to reduce nucleosome stability and cause defects in the DDR in vivo [93]. This clearly demonstrated the importance of H4K77 succinylation to the DDR.

Human flap endonuclease 1 (FEN1) is involved in DNA replication and repair. When cells are stimulated by DNA replication fork blockers such as hydroxyurea, ultraviolet (UV), mitomycin C and camptothecin, succinylation occurs at the K200 site of FEN1 [95]. This promotes the interaction between FEN1 and the Rad9-Rad1-Hus1 complex, restoring normal function to stagnant replication forks. When FEN1 succinylation levels decrease, this leads to the accumulation of DNA damage, which makes the replication forks more sensitive to blockers. In short, FEN1 succinylation serves an important role in the DDR and in the maintenance of genomic stability.

Nuclear phosphoprotein (NPM1), as a member of the H2AX complex, has also been found to undergo acetylation or succinylation at its K27 site, which is highly conserved. Notably, these two modifications are significantly higher in breast cancer tissues than in normal tissues. In addition, cell viability was significantly enhanced after overexpression of NPM1 in BT-549 cells. However, this function was lost after the over-expression of the NPM1-K27R mutant [96]. This strongly suggests that the acetylation and succinylation of NPM1 are involved in the regulation of cell proliferation. Therefore, NPM1 phosphorylation and succinylation modification may represent potential novel targets for the research and treatment of breast cancer, in particular.

### 3.3. Succinylation in Cancer

Lysine succinylation is a recently discovered protein PTM and is dynamic, reversible, and evolutionarily conservative. This process has also been suggested to play a key regulatory role in cancer. For example, the succinylation of M2-pyruvate kinase (PKM2) at K498 has been found to increase PKM2 activity. However, SIRT5 can bind to PKM2, which desuccinylates PKM2 and reduces its activity, contributing to the proliferation of tumor cells. This led to the discovery that the inhibition of SIRT5 or the use of the succinic mimic mutant PKM2(K498E) can inhibit tumor cell proliferation [148]. As a result, SIRT5-mediated desuccinylation of PKM2 has been suggested to contribute to tumorigenesis. In addition, another study demonstrated that S100A10, a member of the calcium-binding cytoplasmic protein family, can bind to CPT1A at K47 and then be succinylated by it; contributing to the proliferation of gastric cancer cells [137]. The succinic acidification mimic mutant S100A10(K47E) has also been used to further verify that CPT1A-mediated S100A10 succinylation increases the invasion and migration of gastric cancer cells [132]. Certainly, it is worth investing more efforts to explore if succinylation could be a novel target for gastric cancer treatment.

## 4. Palmitoylation

### 4.1. S-Palmitoylation

Palmitoylation specifically involves the modification of proteins with palmitic acid. Palmitoylation can be categorized into three types: S-palmitoylation, O-palmitoylation, and N-palmitolation. S-palmitoylation refers to the addition of fatty acyl groups to the cysteine residues of proteins, while O-palmitoylation and N-palmitoylation are the addition of fatty acyl groups to serine residues and to the amino terminus or the epsilon amino group of lysine residues, respectively. Given that S-palmitoylation is the most common form of palmitoylation, this review primarily focuses on this type.

S-palmitoylation often involves the addition of palmitic acid to cysteine residues. Because the thioester bonds formed are very unstable, S-palmitoylation is often reversible and dynamic, and S-palmitoylated proteins can rapidly respond to stimulation by upstream signals [149]. Although some studies examined S-palmitoylation decades ago, little is known about the specific mechanisms underlying palmitoylation and depalmitoylation or about the readers for palmitoylated substrates [150]. Recent studies have shown that palmitoylation is catalyzed by palmitoyl S-acyltransferases (PATs), which in mammals are a family comprising 23 different members [151]. The PAT active site contains the zinc finger domain of Asp-His-His-Cys (DHHC), which is critical to its enzymatic activity [149]. The cysteine residue in the DHHC motif reacts with palmitoyl CoA to form an acyl intermediate, following which the fatty chain is transferred directly to the substrate protein, and CoA is released. Cysteine residues near the DHHC domain are known to be critical for anchoring two zinc atoms for proper enzyme folding and function, but this does not catalyze the transfer of palmitate [150].

#### Depalmitoylation

Protein S-palmitoylation (hereafter referred to as palmitoylation) can produce internal cysteine residues through the thioesterification of hexadecyl saturated fatty acids (known as palmitates), resulting in a dynamic and reversible modification. Deamination of palmitoyl in mammalian cells is performed by cysteine deacetylases, which belong to the serine hydrolase family. The first deaminase to be identified was acyl protein thioesterase (APT) [152], which includes two isomers: APT1 and APT2 [153,154]. However, their localization in cells is different. APT1 is present in both the cytoplasm and mitochondria, while APT2 exists only in the cytoplasm, resulting in differences in their function. For example, in vitro, APT1 can depalmitoylate the G protein α subunit and H-Ras [155]. However, the restriction of APT2 to the cytoplasm in the cell suggests that APT2 selectively inhibits the palmitoylation of cytoplasmic proteins.

**Figure 3:**
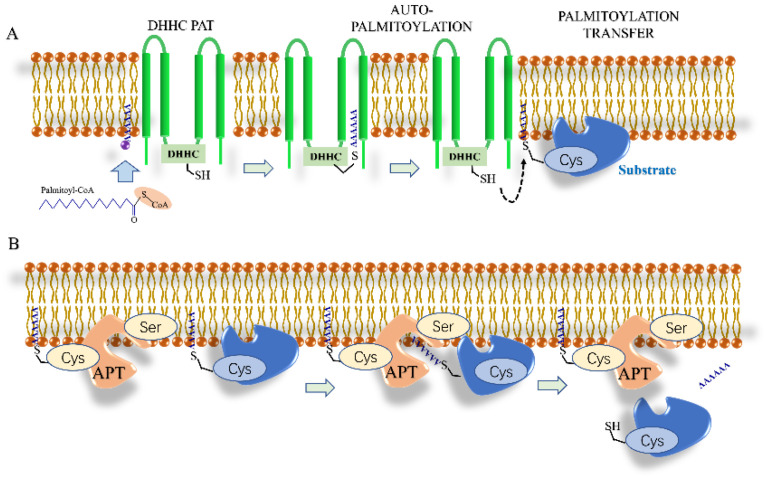
Palmitoylation modification mechanism. (**A**) The process of protein succinylation. (**B**) The process of protein desuccinylation.

An increasing number of studies on palmitoylation of proteins have revealed that APT1 and APT2 are not the only thioesterases in cells. At least 19 members of the mammalian α/β hydrolase domain protein family are thought to have depalmitoylase functions, including ABHD17A, ABHD17B, and ABHD17C depalmitoylases [156]. These proteins contain multiple conserved cysteine residues near their N-terminus, which can regulate the palmitoylation of N-Ras. In addition, palmitoylthioesterase 1 and 2 (PPT1/2) are proteins found in the lysosomes, where they deacylate palmitoylated proteins. The process of palmitoylation is depicted in Figure 3.

### 4.2. Palmitoylation in the DDR

Cancer is primarily driven by the accumulation of genetic mutations (i.e., by gain-of-function mutations in oncoproteins or the loss of tumor suppressor functions). During the DDR, different types of DNA damage are detected, and corresponding repair modes are selected to fix damaged DNA and remove any irreparable DNA. There is evidence to suggest that protein palmitoylation and PATs are involved in both the occurrence and development of cancer via the regulation of the DDR [149]. For example, zDHHC gene expression is altered in a number of tumor tissues [157]. In MEF cells, deletion of zDHHC 16 gene encoding palmitoyltransferase affects the formation of DNA damage foci, the activation of ATM, and the induction and activation of p53. This suggests that zDHHC 16, as a PAT, plays an important regulatory role in the DDR process, and this may represent a novel avenue for cancer treatment [158].

Rap1-interactingfactor1 (Rif1), a telomere-binding protein discovered for the first time in *Saccharomyces cerevisiae*, can weaken the excision of DNA ends in the event of telomere dysfunction. Notably, the cysteine residues C466 and C473 in the conserved N-terminal domain of Rif1 have been found to be S-acylated by the DHHC family palmitoyltransferase Pfa4. S-acylation of Rif1 promotes the recruitment and accumulation of Rif1 at DSBs, which weakens the excision of DNA ends and promotes the repair of non-homologous terminal junctions. These findings confirm the contribution of S-acylated Rif1 to DSB repair [97].

Epidermal growth factor receptor (EGFR) mutations are common in pleomorphic glioblastoma (GBM), for which there is currently no effective treatment. EGFR mutations in GBM are known to result in the inactivation of the ZDHHC16/SETD2/H3K36me3 signaling axis. However, ZDHHC16 expression in GBM is weaker than that in normal brain tissue, which promotes activation of p53 and leads to cell cycle arrest at the G1/S checkpoint. In addition, after ionizing radiation-induced DNA damage, SETD2 palmitoylation and H3K36 methylation in the DDR signaling pathway were decreased in EGFR-amplified GBM, resulting in p53 inactivation and G1/S checkpoint stagnation. Simultaneously, the depalmitoylation inhibitor PalmB was identified as a potential novel target for adjuvant therapy in patients with GBM [159]. However, research concerning palmitoylation in the context of the DDR remains limited, and more extensive and in-depth studies are required to characterize these pathways in full.

### 4.3. Palmitoylation in Cancer

Multiple proteins that undergo palmitoylation have been identified in mammals, and these palmitoylated proteins are often closely associated with cancer. Some of these proteins are mono-palmitoylated, but others are palmitoylated in multiple locations [160]. In addition, some proteins are spontaneously palmitoylated [161]. However, the majority of protein palmitoylation is catalyzed by the PAT family, which contains zinc finger structures (ZDHHCs). PAT and APT enzyme function is known to be associated with cancer, and the regulation of these enzymes has been suggested as a potential target for cancer treatment [162,163,164].

Low ZDHHC2 expression has been identified in both gastric adenocarcinoma and highly metastatic mouse colon adenocarcinoma clones, but its candidate substrates and potential underlying anti-tumor mechanisms remain unclear [165,166]. In addition, the expression of ZDHHC17 (also known as HIP14) is upregulated in breast and colon cancer [167]. In HEK293 cells, when ZDHHC14 is overexpressed, the formation of xenograft tumors is inhibited while cell apoptosis is promoted [168]. Furthermore, in lung cancer, although ZDHHC5 expression levels have little to do with overall survival, the inhibition of ZDHHC5 expression results in significantly decreased cell proliferation, migration, and xenograft growth [169]. The overexpression of ZDHHC5 often indicates a poor prognosis in glioma [170].

These results suggest that the regulation of ZDHHC enzymes is common to multiple types of cancer, and targeted regulation of protein palmitoylation may represent a promising new avenue for anticancer therapy [149].

## 5. N-myristoylation

### 5.1. N-myristoylation

N-myristoylation is a process characterized by the covalent linking of a 14-carbon saturated fatty acid, myristic acid, to the N-terminal glycine residue of a protein. Unlike palmitoacylation, this process is irreversible, resulting in permanent changes to the physiological functions of proteins. These changes can include cellular localization, plasma targeting, and subcellular tracking. Protein N-myristoylation has been found to occur in multiple diseases, and as a result, this PTM has received widespread attention [171].

As a member of the GCN5-related N-acetyltransferase (GNAT) superfamily [172,173,174], N-myristoyltransferase (NMT) is responsible for myristic acid transfer during N-myristoylation. At present, only one NMT subtype has been identified in lower eukaryotes, such as *Saccharomyces cerevisiae* and *Drosophila melanogaster*. However, NMT1 and NMT2 isozymes have been found in a range of mammalian species, including humans and mice [175,176,177]. Although these isozymes are encoded by different genes, they display ~77% sequence homology at the N-terminus, unique substrate specificity, and different physiological functions. In addition, each isozyme has a highly conserved catalytic domain, indicating the importance of this conserved sequence. Previous studies have identified four NMT1 subtypes and two NMT2 subtypes in humans, which are produced via mRNA splicing variants from different reading frames [178]. Furthermore, these subtypes primarily differ in the N-terminus, which may be the reason for the specificity shown in terms of intracellular localization and substrate selection. NMT1 expression levels are similar to those of NMT2 in normal mouse tissues but higher than that of NMT2 in embryos [171]. NMT1 knockdown can seriously damage the differentiation ability of embryonic stem cells, which shows that NMT1 plays an important role in embryogenesis. Taken together, these results indicate that NMT1 exists in a range of different species, and its degree of sequence conservation suggests that it is involved in basic physiological processes.

#### Demyristoylation

Sirtuin2 (SIRT2) is a human lysine deacetylase that belongs to the sirtuin protein family [175,179], but it also serves an additional function as a demyristoylase. Studying the crystalline structure of SIRT2 revealed that it contained a hydrophobic pocket in a complex with thiomyristoyl peptides, which could hold a myristoyl group. This hydrophobic acyl pocket is similar to others found in different sirtuins, including SIRT1, SIRT3, and SIRT6. SIRT6 has also been reported to catalyze the activity of fatty acid deacylase [180]. In addition, despite close structural similarities between the hydrophobic pockets found in SIRT1, SIRT2, and SIRT3, the pockets found in SIRT1 and 3 differ in function from SIRT2, indicating the importance of different long-chain fatty acids involved in deacylation.

**Figure 4:**
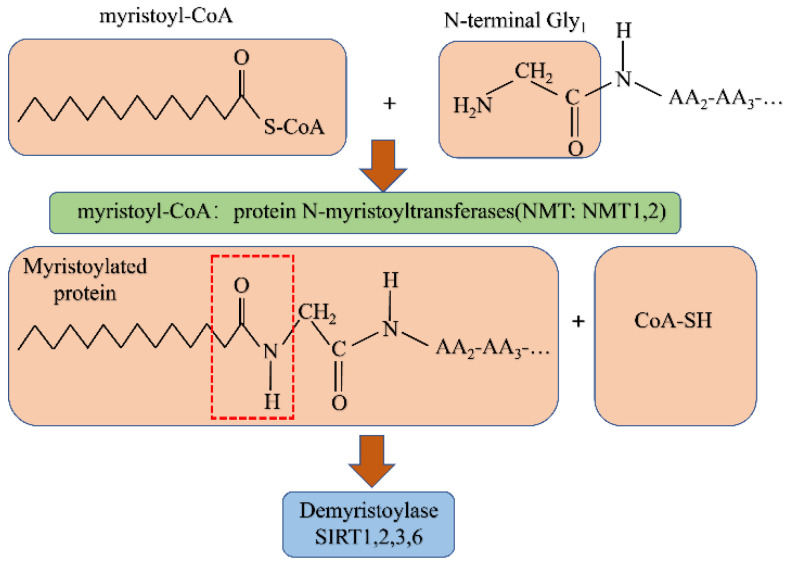
Cardamom acylation modification mechanism.

Shigella virulence factor (IpaJ) is an irreversible de-myristic enzyme that can break the peptide bond formed between N-cardamom acylated glycine-2 and Asn-3 in certain N-cardamom acylated proteins [181]. Because the actions of demyristoylases are irreversible, this provides a novel avenue for the study of the regulatory role of N-myristoylation in human health and disease. The myristoylation modification mechanism is detailed in Figure 4.

### 5.2. N-Myristoylation in the DDR

Finkel-Biskis-Reilly mouse osteosarcoma virus (FBRv-Fos) is a retroviral homologue of the c-fos proto-oncogene and inhibits the response to ionizing radiation-associated DNA damage. As a result, the survival rate of cells exposed to ionizing radiation decreases significantly once FBRv-Fos is expressed [182]. Additional studies revealed that the FBRv-Fos protein could be modified by myristoylation [183]. Notably, this study also demonstrated that retroviral oncogenes are involved in the DDR and that the myristoylation of these oncogenes increases genomic instability. However, few studies concerning the effect of myristoylation in the DDR have been conducted, and more research is required to explore its functional importance [98].

### 5.3. N-myristoylation in Cancer

NMT expression has been observed in multiple types of cancer, and a number of myristoylated proteins are involved in the regulation of cell proliferation and death signaling during cancer [150]. Therefore, NMTs and N-myristoylation are considered promising targets for cancer therapy.

N-myristoylation is known to increase the occurrence of prostate cancer in a Src family-mediated manner [184]. Myristoyl-CoA analog B13 and its derivative LCL204 are inhibitors of NMT1 activity and Src myristoylation and compete for myristoyl-CoA binding sites, effectively inhibiting Src family kinase-mediated cancer [185]. In humans, the organic palladium compound Tris (dibenzylideneacetone) dipalladium (Tris DBA), a novel NMT1 inhibitor, has been found to inhibit both NMT1 activities and reduce its overall expression [186,187]. Tris DBA has also been shown to inhibit proliferation-associated signaling pathway proteins such as Akt, MAPK, and STAT-3 in melanoma [188]. Furthermore, allosteric inhibitors associated with myristoylation, such as GNF2, GNF5, and ABL001, differ from traditional inhibitors that bind to ATP pockets. Instead, these selective target myristoyl-binding pockets in the C leaves of the Abl kinase domain. At present, ABL001 and TKI (nilotinib, dasatinib, and imatinib) are in phase I clinical trials for the treatment of patients with chronic myeloid leukemia (https://clinicaltrials.gov/ct2/show/NCT02081378, accessed on 6 November 2022).

Although genomic changes and somatic mutations in NMT1 and NMT2 have been reported in the Cancer Genome Map (TCGA), the specific regulatory mechanisms of these mutations in cancer remain unclear (https://www.cbioportal.Org/, accessed on 6 November 2022). Additional reports have suggested that patients with high NMT1 or NMT2 expression are more likely to have a poor prognosis [177,189]. Therefore, it seems likely that a more systematic study of N-myristoylation will help identify its functions in tumorigenesis [171].

## 6. Crotonylation

### 6.1. Crotonylation

Lysine crotonylation (Kcr) was first discovered in 2011 [190]. At present, a variety of histone and non-histone lysine residues have been found to undergo crotonylation [21,191]. Notably, the majority of histone Kcr sites identified thus far are located in transcriptional initiation sites (TSSs) and enhancer regions, indicating that histone Kcr may play an important role in gene regulation [190]. Histone Kcr is associated with a range of physiological and pathological processes, including differentiation, tissue injury, neurodegenerative diseases, and tumorigenesis. In addition, alterations in histone Kcr have been detected in liver cancer, gastric cancer, lung cancer, and renal cell carcinoma [192].

#### 6.1.1. Writers for Crotonylation

Histone crotonyltransferases include histone acetyltransferases (HATs) such as p300, GCN5, Tip60, and MOF, with p300 being the first crotonyltransferase to be found [193]. In vitro studies have demonstrated that the knockdown of p300 expression by siRNAs leads to decreased H3K18 crotonylation levels [194]. However, due to the presence of a limiting aliphatic back pocket, the crotonyl transferase activity of p300 is significantly lower than its acetyltransferase activity. Thus, the catalytic efficiency of p300 may need to be improved by other auxiliary factors. For example, MOF has been reported to catalyze crotonylation at histone H3 lysine residues 4, 9, 18, and 23 and histone H4 lysine residues 8 and 12 in HeLa cells [195]. GCN5 can catalyze crotonylation in budding yeast of lysine residues at sites 9, 14, 18, 23, and 27 of histone H3 [196], and Esa1, a MOF homolog found in humans, also catalyzes the crotonylation of lysine residues 5, 8, 12 and 16 in histone H4 [196].

#### 6.1.2. Erasers for Crotonylation

The sirtuin family, also known as class III HDACs, were the first histone deacetylases to be found. SIRT1, 2, and 3 are also considered “erasers” of crotonylation, which remove Kcr markers from histones [197]. In addition, class I HDACs such as HDAC1, HDAC2 and HDAC3 also mediate histone decrotonyltion and seem to have stronger regulatory effects than the sirtuins [198]. HDAC1 is known to exert decrotonylation activity at multiple sites, including H3K4, H3K9, H3K23, H4K8, and H4K12. However, HDAC2 and 3 only regulate the decrotonylation of the H3K23 site. In addition, SIRT1 only serves as an eraser at the H3K9Cr and H4K8Cr sites [198].

#### 6.1.3. Readers for Crotonylation

The reader module is responsible for the recognition of histone modification during epigenetic regulation. The earliest histone crotonylation reader to be discovered was the Yeats domain, which is highly conserved and is found in organisms as disparate as humans and yeast. Members of the Yeats family participate in histone modification, histone deposition, transcriptional regulation, and chromatin remodeling through the formation of a variety of important complexes [199]. The transcription factor complex TFIID and TFIIF [200], the chromatin remodeling complex RSC, SWI/SNF, and INO80, and the histone acetyltransferase complex NuA3 all contain Yeats domains [200]. In addition, the Taf14 Yeats domain is known to read H3K9Cr. The Yeats domain of YEATS2 binds to crotonylated proteins through an end-open aromatic sandwich pocket. The binding ability of this protein to H3K9Cr, H3K12Cr, H3K23Cr, and H4K4Cr is weak but is very strong in H3K27Cr [201]. In contrast, the mechanism of AF9 YEATS differs from those of the aforementioned proteins. Instead, it binds closely to H3K9Cr, H3K18Cr, and H3K27Cr through the vesicles formed by its L1, L4, and L6 rings [202].

**Figure 5:**
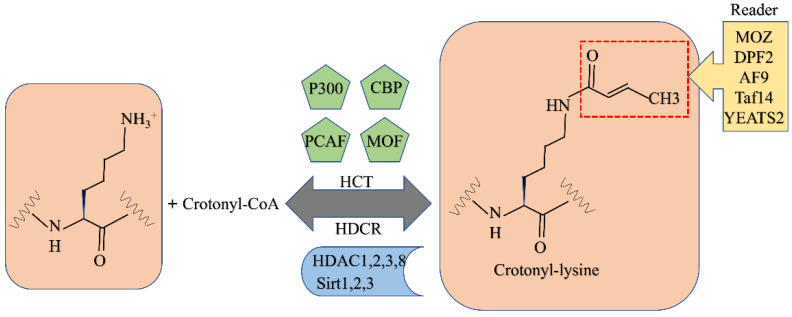
Crotonylation modification mechanism.

The double PHD finger (DPF) domain was the second histone crotonylation reader to be identified. The DPF domains of human MOZ (also known as KAT6A) and DPF2 (also known as BAF45d) can bind to two acetylated histones, which recognize H3K14Cr through a hydrophobic “dead-end” pocket [203]. The crotonylation process is detailed in Figure 5.

### 6.2. Crotonylation in the DDR

Histone crotonylation has recently been revealed to serve an important regulatory role in the DDR process, and HDACs have both Kac and Kcr enzymatic activities. Therefore, it is necessary to selectively target one of these activities to clarify how these different modifications regulate different aspects of the DDR [204]. When cells were exposed to ultraviolet radiation, ionizing radiation, laser microirradiation, or DNA-damaging agents such as etoposide, HDAC-mediated H3K9cr levels at sites of DNA damage decreased to various degrees [204]. However, RPA1 Kcr levels were upregulated, and this was negatively regulated by CDYL1 [205]. This occurs via the following mechanism: Kcr modification of RPA1 enhances the interaction between RPA1 and single-stranded DNA and resection machinery components, which aids the survival of cells following DNA damage [21]. In addition, although CDYL can reduce RPA1 Kcr levels, it is not the sole regulator. HCT and/or HDCRs (both class I and III HDACs have been classified as histone decrotonaylases (HDCRs)) also exert regulatory influences here. Thus, during DNA damage, decreased RPA1 Kcr levels are likely to be the result of multiple factors [21].

However, only a few studies concerning the involvement of crotonylation in the DDR have been conducted, and more work will be needed to fully characterize its involvement in this process.

### 6.3. Crotonylation in Cancer

It is worth noting that crotonylation levels are decreased in liver cancer, renal cell carcinoma, and gastric cancer but are increased in lung, esophagus, thyroid, colon, and pancreatic cancer [206]. The crotonylation of proteins has also been reported in all eight types of cervical cancer [207]. This suggests that the regulatory effects of crotonylation are heavily dependent on the type of cancer. Furthermore, Kcr is also known to regulate the movement and proliferation of hepatocellular carcinoma cells [206]. In short, since there is a general lack of data concerning protein crotonylation, more research to explore the involvement of crotonylation in cancer is required.

## 7. Conclusions

The DDR represents a complex network of different signaling pathways that are activated in response to both exogenous and endogenous DNA damage. This involves a variety of cellular processes, including cell cycle checkpoints, the repair of damaged DNA, and the apoptosis of cells where DNA cannot be fixed. In this way, the genome is able to properly respond to DNA damage and maintain genomic stability, which, if defective, can lead to conditions such as cancer [48].

Protein PTMs such as phosphorylation, acetylation, methylation, and ubiquitination all play important roles in the DDR. However, in recent years, the development of highly sensitive and efficient mass spectrometry and proteomics has also expanded our understanding of protein acylation. This paper reviews five important classes of protein-lipid modifications: acetylation, succinylation, palmitoylation, N-myristoylation, and crotonylation. Interestingly, some PTM writers, such as p300/CBP, have many kinds of enzyme activities. p300/CBP not only exerts its well-characterized acetyltransferase activity but also catalyzes the propionylation (Kpr) [208], butyrylation (Kbu) [209], and Kcr [210] of histone lysine. Further analysis of p300 activity and kinetics found that, although p300 can catalyze these acylation reactions, the reaction rate decreases as the acyl chain increases in length [211]. While GCN5 can catalyze Kpr and Kbu, its catalytic activity for Kpr is higher [212], and myristoyl transferases 1 and 2 also belong to the GCN5 acetyltransferase family [213]. Likewise, partial erasers of these processes are also not unique. For example, SIRT1/2/3 [214,215] not only functions as a deacetylase but also as a depropionylase and butyrylase. While SIRT5 desmalonylase and deglutaraylase activities are relatively high, their deacetylase activity is relatively low [216]. Similarly, bromodomains, as a typical reader of histones, mainly bind to Kac but can also bind to Kpr and Kbu, albeit more strongly to the former [217]. However, the binding ability of the DPF1/2/3 domain to Kcr is stronger than that to Kac, mainly due to hydrophobic encapsulation and coordinated hydrogen bonding [203]. In short, the exact mechanisms of these writers, erasers, and readers are not clearly characterized at present, and this needs to be explored in greater depth.

The molecular weight and charge of lipid metabolite PTMs mean that they can alter a variety of chemical and structural properties of proteins. For example, protein function can be affected by changing the conformation and subcellular localization of the protein, changing the stability of the protein, or changing its interactions with other proteins, and different types of PTMs can also compete. Therefore, in order to cope with DNA damage, lipid metabolism PTMs and other types of PTMs function both independently and synergistically, ensuring that proteins are recruited to DNA damage sites in the right order and at the right position. However, more in-depth research is needed to decipher the network relationship between different protein PTMs and to use the dynamic network relationships between protein PTMs to determine, explain or predict the response of cells to different types of DNA damage. In this way, more targets and pathways for cancer treatment can be explored.

The extensive and in-depth research conducted thus far on epigenetic modifications and their effects on proteins, these modifications have gradually become a target for cancer treatment. The majority of current clinical experiments are based on mutations of target proteins in cancer, but in some tumors, these target proteins may be epigenetically modified rather than mutated. Thus, screening for mutations in target proteins is unlikely to be sufficient. At present, the best and most effective anti-tumor methods target multiple oncogenic pathways at the same time, and the combination of epigenetic target protein drugs with more traditional chemotherapy drugs would likely exert beneficial therapeutic effects. Although epigenetic drugs have limitations, their potential lends great incentives to researchers and the pharmaceutical industry to explore and develop more effective target drugs for cancer treatment. Ultimately, maintenance of the integrity and stability of the genome using such drugs may reduce the occurrence and development of cancer.
